# Bilirubin Distribution in Plants at the Subcellular and Tissue Levels

**DOI:** 10.1093/pcp/pcae017

**Published:** 2024-02-27

**Authors:** Kazuya Ishikawa, Yutaka Kodama

**Affiliations:** Center for Bioscience Research and Education, Utsunomiya University, Tochigi 321-8505, Japan; Graduate School of Medicine, Dentistry, and Pharmaceutical Sciences, Okayama University, Okayama 700-8530, Japan; Center for Bioscience Research and Education, Utsunomiya University, Tochigi 321-8505, Japan

**Keywords:** *Arabidopsis thaliana*, Bilirubin, Live imaging, *Nicotiana benthamiana*, UnaG

## Abstract

In heterotrophs, heme degradation produces bilirubin, a tetrapyrrole compound that has antioxidant activity. In plants, heme is degraded in plastids and is believed to be converted to phytochromobilin rather than bilirubin. Recently, we used the bilirubin-inducible fluorescent protein UnaG to reveal that plants produce bilirubin via a non-enzymatic reaction with NADPH. In the present study, we used an UnaG-based live imaging system to visualize bilirubin accumulation in *Arabidopsis thaliana* and *Nicotiana benthamiana* at the organelle and tissue levels. In chloroplasts, bilirubin preferentially accumulated in the stroma, and the stromal bilirubin level increased upon dark treatment. Investigation of intracellular bilirubin distribution in leaves and roots showed that it accumulated mostly in plastids, with low levels detected in the cytosol and other organelles, such as peroxisomes, mitochondria and the endoplasmic reticulum. A treatment that increased bilirubin production in chloroplasts decreased the bilirubin level in peroxisomes, implying that a bilirubin precursor is transported between the two organelles. At the cell and tissue levels, bilirubin showed substantial accumulation in the root elongation region but little or none in the root cap and guard cells. Intermediate bilirubin accumulation was observed in other shoot and root tissues, with lower levels in shoot tissues. Our data revealed the distribution of bilirubin in plants, which has implications for the transport and physiological function of tetrapyrroles.

## Introduction

Bilirubin (bilirubin IXα) is a catabolite of heme, an essential biomolecule consisting of a tetrapyrrole ring containing an iron cation. In heterotrophic organisms, heme is synthesized in the mitochondria and then distributed throughout the cell, where it binds to various proteins as a cofactor. Heme-containing proteins (hemoproteins) play essential roles in numerous cellular functions, including oxygen transport (hemoglobin), electron transfer (mitochondrial respiratory complexes) and detoxification (cytochrome P450 enzymes and catalases). However, free heme released by the breakdown of hemoproteins is a powerful oxidant that increases cellular oxidative stress; therefore, its intracellular levels are tightly controlled via heme degradation (Chiabrando et al. 2014; Kumar and Bandyopadhyay, 2005; Shepherd et al. 2013).

In heme degradation, heme oxygenase oxidizes heme to the linear tetrapyrrole biliverdin, which is reduced to bilirubin by an NADPH-dependent biliverdin reductase. Although a high level of bilirubin is toxic and can cause brain damage (Vítek and Ostrow 2009), bilirubin is also a strong antioxidant. Therefore, a mild increase in bilirubin levels prevents oxidative damage to cells and reduces the risk of diseases such as arterial hypertension and diabetes (Jangi et al. 2013, Mancuso 2017, Yao et al. 2019). Furthermore, bilirubin has attracted considerable attention because of its functions as a signaling molecule and hormone (Creeden et al. 2021, Vítek and Tiribelli 2021).

Heme is also essential for plant growth, playing various roles as a cofactor for proteins, including in plant-specific complexes such as the photosystems, where it is a component of the cytochrome proteins required for electron transport. Unlike in heterotrophic organisms, in plants, heme is biosynthesized in plastids, where the enzyme ferrochelatase, which introduces an iron cation into the tetrapyrrole ring, is localized. Plants were believed to convert biliverdin to phytochromobilin, the chromophore of phytochrome (Kohchi et al. 2001), rather than to bilirubin (Lagarias et al. 1997, Montgomery et al. 2001). In our previous study, we used UnaG, a bilirubin-inducible fluorescent protein (Kumagai et al. 2013), to demonstrate that bilirubin is produced in chloroplasts by a non-enzymatic reaction of biliverdin with NADPH generated by photosystem I (Ishikawa et al. 2023). Photosystem I converts light energy into reducing power in the form of NADPH, but under conditions of strong light, excess NADPH is generated. This excessive NADPH reduces oxygen, producing superoxide and other reactive oxygen species (ROS) (Foyer and Hanke 2022). Therefore, we proposed that the non-enzymatic reaction of biliverdin with NADPH plays a role in attenuating oxidative stress in chloroplasts by producing bilirubin as an antioxidant while consuming excess NADPH (Ishikawa et al. 2023).

Understanding the transport and distribution of metabolites is important for elucidating their physiological functions, but the transport and distribution of heme and its degradation products in plant cells are largely uncharacterized. In animal cells, ATP-binding cassette (ABC) transporters and major facilitator superfamily (MSF) transporters are known to transport heme (Khan and Quigley 2011), but these two types of heme transporters have not been identified in plants. Tryptophan-rich sensory protein (TSPO) was reported to be involved in heme transport in *Arabidopsis* (*Arabidopsis thaliana*) cells (Vanhee et al. 2011). TSPO is localized in the endoplasmic reticulum (ER) and Golgi apparatus, suggesting the existence of a transporter other than TSPO that exports heme from chloroplasts, the site of heme synthesis. Even less information is available regarding biliverdin distribution, and to our knowledge, no factors involved in its transport have been identified in plants. Phenotypic analysis of *Arabidopsis* plants exhibiting ectopic expression of rat biliverdin reductase implies that biliverdin is present in the cytosol in addition to the plastids (Montgomery et al. 2001). Given this lack of information on the distribution of heme and its degradation products, our goal was to investigate the distribution of bilirubin in plants using UnaG. Here, we report the distribution of bilirubin in *Arabidopsis* from the organelle level to the tissue level, and provide insight into the function of bilirubin and the transport system of tetrapyrroles.

## Results

### Bilirubin is mainly localized to the stroma in chloroplasts

**Fig. 1:**
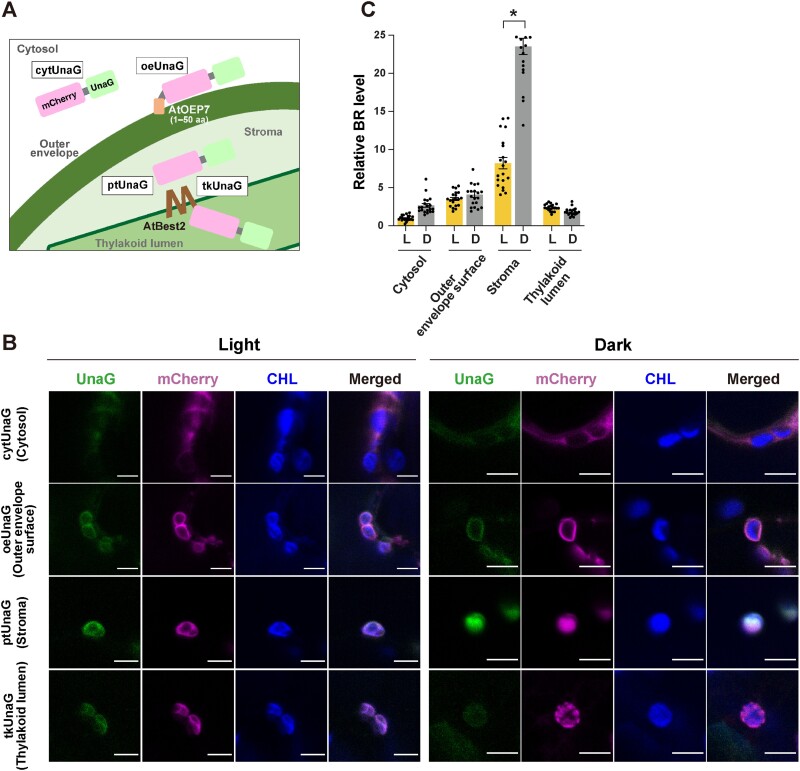
High bilirubin levels in the chloroplast stroma. (A) Schematic showing targeting of UnaG to the cytosol (cytUnaG), chloroplast outer envelope surface (oeUnaG), stroma (ptUnaG) or thylakoid lumen (tkUnaG). oeUnaG and tkUnaG are fused with the N-terminal sequence (50 aa residues) of *Arabidopsis* OUTER ENVELOPE PROTEIN7 (AtOEP7) and *Arabidopsis* BESTROPHIN-LIKE PROTEIN2 (AtBest2), respectively, and the mCherry–UnaG portion of oeUnaG and tkUnaG is exposed to the cytosol and thylakoid lumen. (B) UnaG fluorescence, mCherry fluorescence, chlorophyll autofluorescence and a merged image of the three in *N. benthamiana* epidermal cells transiently expressing cytUnaG, oeUnaG, ptUnaG or tkUnaG after 48 h of incubation in the light (L) or in darkness (D). Bars, 5 µm. (C) Quantification of bilirubin (BR) levels in the cellular regions shown in (B). Cytosol bilirubin levels in light-treated leaves were set to 1. *n* = 20. Data are shown as means ± SD; *P < 0.01, using a two-tailed Student’s *t*-test.

To analyze where bilirubin accumulates in the chloroplasts, we transiently expressed mCherry–UnaG targeted either to the chloroplast outer envelope surface (oeUnaG), the stroma (ptUnaG; Ishikawa et al. 2023) or the thylakoid lumen (tkUnaG) (**Fig. 1A**), and investigated the bilirubin levels in both light and dark conditions. mCherry was fused to UnaG as an internal control and free mCherry–UnaG (cytUnaG) was expressed as a second control. We measured UnaG and mCherry fluorescence intensities to quantify bilirubin levels in each of the targeted subcompartments (**Supplementary Figure S1**). In light, bilirubin levels were 3.51, 8.21 and 2.37 times higher in the outer envelope surface, stroma and thylakoid lumen, respectively, than in the cytosol (**Fig. 1B**, **C**). In dark, only stromal bilirubin levels were significantly elevated to 2.86 times to those seen in the light. Furthermore, we noticed that UnaG displayed an uneven fluorescence pattern in the stroma. Taken together, these results suggest that, in both light and dark conditions, bilirubin accumulates mainly in the stroma, where the NADPH-producing systems reside.

### Bilirubin preferentially accumulates in plastids in leaf and root cells

**Fig. 2:**
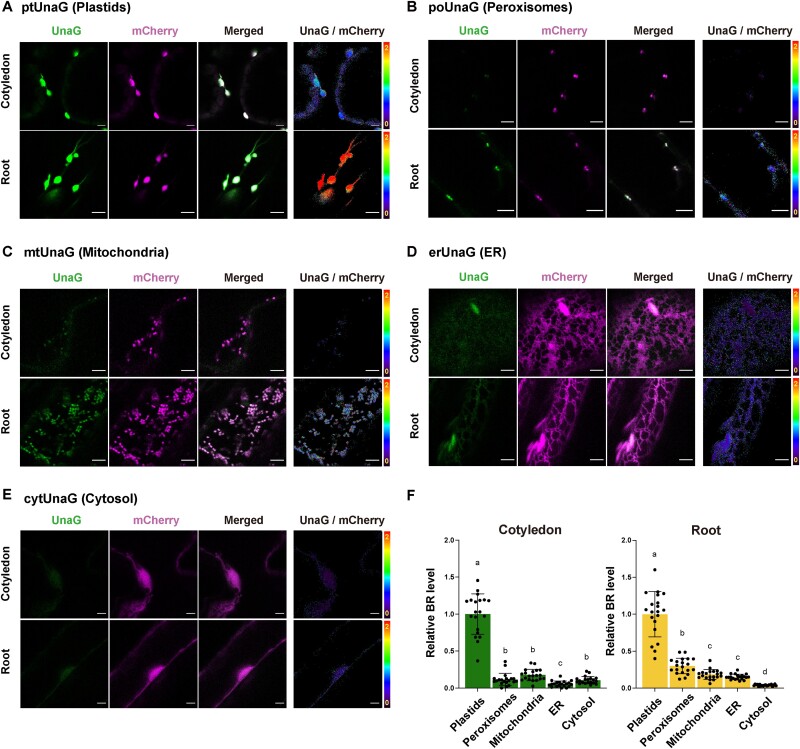
Bilirubin visualization at the organelle level reveals preferential accumulation in plastids. (A–E) Fluorescence of UnaG and mCherry when targeted to different organelles. Epidermal cells of cotyledons or roots (elongation zone) in 7-d-old *Arabidopsis* seedlings expressing ptUnaG (A), poUnaG (B), mtUnaG (C), erUnaG (D) or cytUnaG (E). The rightmost panels in each set are ratiometric images (UnaG/mCherry) with the color key. Bars, 5 µm. (F) Quantification of organellar bilirubin (BR) levels in cotyledons and roots. Plastid bilirubin levels were set to 1. *n* = 20. Data are shown as means ± SD; different letters indicate significant difference, *P* < 0.05, using Tukey’s multiple comparison test.

To assess the subcellular distribution of bilirubin, we generated transgenic *Arabidopsis* plants expressing various constructs encoding mCherry–UnaG targeted to plastids, peroxisomes, mitochondria, the ER and the cytosol (ptUnaG, poUnaG, mtUnaG, erUnaG and cytUnaG, respectively). As a marker for the bilirubin level in plastids, we used a stromal marker ptUnaG, which shows the highest bilirubin level in plastids (**Fig. 1**). We imaged and measured the UnaG and mCherry fluorescence intensities to quantify organellar bilirubin levels in various organelles and tissues of cotyledon and root cells (**Fig. 2A-F**, **Supplementary Figure S2**). In cotyledon epidermal cells, relative mean bilirubin levels to plastid levels reached 0.11, 0.17, 0.15 and 0.10 in peroxisomes, mitochondria, the ER and the cytosol, respectively (**Fig. 2F**). We found a similar trend in roots: relative bilirubin levels to plastid levels in peroxisomes, mitochondria, the ER and the cytosol were 0.29, 0.18, 0.15 and 0.03, respectively (**Fig. 2F**). These results demonstrated that bilirubin accumulates in different organelles at different concentrations and preferentially accumulates in plastids, both in cotyledons and roots. This is reasonable because the heme oxygenases are localized in plastids (Gisk et al. 2010). In addition, relatively high levels of bilirubin were found in peroxisomes in roots. As peroxisomes are known to have relatively high concentrations of NADPH (Lim et al. 2020), bilirubin may also be produced in peroxisomes.

### Increased bilirubin production in plastids affects bilirubin accumulation in peroxisomes

**Fig. 3:**
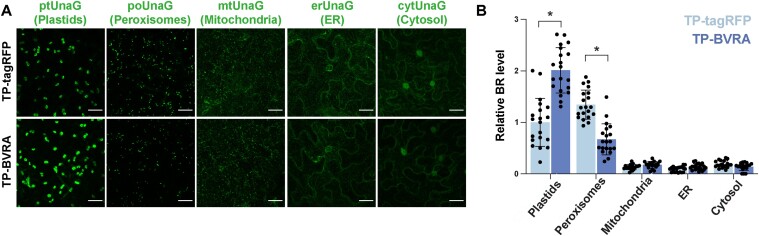
Increased bilirubin production in chloroplasts decreases the bilirubin level in peroxisomes. (A) Either TP-tagRFP (control) or TP-BVRA was transiently co-infiltrated with constructs expressing ptUnaG, poUnaG, mtUnaG, erUnaG or cytUnaG in epidermal cells of *N. benthamiana* leaves. Bars, 25 µm. (B) Quantification of bilirubin (BR) levels in each organelle. Plastid bilirubin levels for TP-tagRFP accumulation were set to 1. *n* = 20. Data are shown as means ± SD; *P < 0.01, using a two-tailed Student’s *t*-test.

Although bilirubin is lipid-soluble and membrane-permeable in mammalian cells (Ives and Gardiner 1990, Bulmer et al. 2008), bilirubin concentrations varied among organelles in plant cells (**Fig. 2**), raising the possibility that bilirubin cannot passively diffuse through plant organellar membranes. Therefore, we measured organellar bilirubin levels using ptUnaG, poUnaG, mtUnaG, erUnaG or cytUnaG when bilirubin biosynthesis in chloroplasts was upregulated. We increased bilirubin biosynthesis through the heterologous overexpression of a construct encoding a version of rat biliverdin reductase A targeted to plastids (TP-BVRA) in *N. benthamiana* (Lagarias et al. 1997, Montgomery et al. 2001). Plastid-targeted tagRFP (TP-tagRFP) was used as a control, and the relative bilirubin level in each organelle to the plastid bilirubin level in cells expressing TP-tagRFP was calculated. As expected, TP-BVRA expression increased the level of plastid bilirubin relative to that seen in the cell-expressing TP-tagRFP (**Fig. 3A**, **B**). Conversely, bilirubin levels in peroxisomes significantly decreased when TP-BVRA accumulated in plastids, suggesting transfer between these organelles. However, TP-BVRA accumulation in plastids did not significantly alter bilirubin levels in mitochondria, the ER or the cytosol. Increasing the bilirubin levels in plastids therefore did not increase bilirubin levels in other organelles, suggesting that bilirubin cannot freely diffuse through organellar membranes in plant cells. In contrast, higher bilirubin levels in plastids lowered bilirubin levels in peroxisomes, implying a complex exchange of bilirubin or its substrate biliverdin between plastids and peroxisomes.

### Bilirubin preferentially accumulates in root epidermal cells

**Fig. 4:**
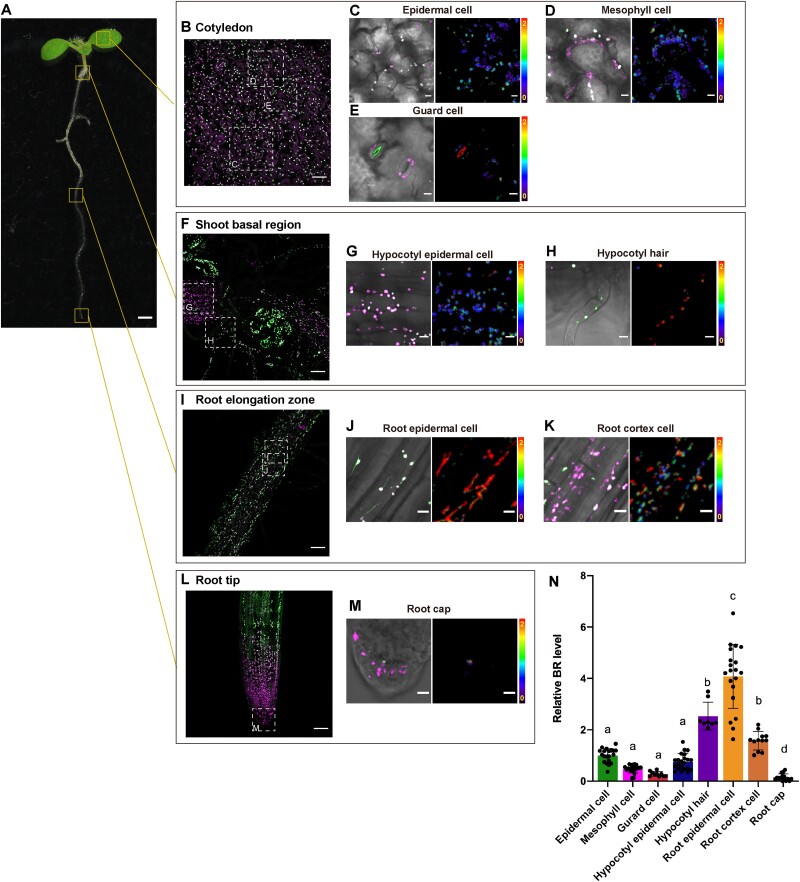
Bilirubin shows uneven distribution among different tissues. (A–M) Expression of ptUnaG in 7-d-old *Arabidopsis* seedlings. (A) A sample seedling showing the regions analyzed. The images in panels B, F, I and L show UnaG and mCherry fluorescence in cotyledons (B), the basal region of the shoot (F), the root elongation zone (I) and the root tip (L). Bars, 50 µm. The areas indicated by dotted squares in these panels are magnified in panels C–E, G and H, J and K and M. In each magnified set, the left panels are merged images (UnaG, mCherry and brightfield) and the right panels are ratiometric images (UnaG/mCherry) with the color key. Bars, 10 µm. (N) Quantification of bilirubin (BR) levels in each tissue, calculated on the basis of the data in **Supplementary Figure S3**. *n* = 20 epidermal cells, 20 mesophyll cells, 15 guard cell, 22 hypocotyl epidermal cells, 10 hypocotyl hairs, 20 root epidermal cells, 12 root cortex and 15 root cap. Bilirubin levels in the cotyledon epidermal cells were set to 1. Data are shown as means ± SD; different letters indicate significant differences at *P* < 0.05, using ANOVA and Tukey’s multiple comparison test.

To determine and quantify the cell- and tissue-specific distribution of bilirubin, we measured bilirubin levels in plastids in various tissues in transgenic *Arabidopsis* plants accumulating ptUnaG (**Fig. 4A**). Although we detected UnaG fluorescence throughout the seedlings, the fluorescence intensity varied among tissues (**Fig. 4B**–**M**). Therefore, we measured UnaG and mCherry fluorescence intensity in plastids for each tissue (**Supplementary Figure S3A–S3D**), and compared bilirubin levels in each tissue with that in leaf epidermal cells. Bilirubin levels in mesophyll cells and root cortex cells were normalized by using mCherry fluorescence intensities because the fluorescence was attenuated by sample thickness. In cotyledons (**Fig. 4B**), relative mean bilirubin levels in mesophyll cells and guard cells were 0.47 and 0.27, respectively (**Fig. 4C**–**E** and **N**). In the shoot basal zone (**Fig. 4F**), relative bilirubin levels were 0.74 in hypocotyl epidermal cells and 2.53 in hypocotyl hairs, the hair-like structures growing from the base of the hypocotyl (**Fig. 4G**, **H** and **N**). In the root elongation zone (**Fig. 4I**), relative bilirubin levels were 1.57 in root cortex cells and reached their highest levels among all tissues in root epidermal cells, with relative levels of 4.08 (**Fig. 4J**, **K** and **N**). Conversely, we detected much lower bilirubin levels (0.13) in the root cap (**Fig. 4L-****N**). Collectively, these results indicated that bilirubin is most abundant in plastids in the root elongation zone and quite low in abundance in root cap amyloplasts and guard cell chloroplasts.

## Discussion

In the present study, we analyzed the distribution of bilirubin in plants from the organelle level to the tissue level. To the best of our knowledge, this is the first study to demonstrate the distribution of a tetrapyrrole in plants using live imaging, providing important insights into the biosynthesis and transport of tetrapyrroles. UnaG, which binds to bilirubin with an extremely low dissociation constant, stably emits fluorescence (Kumagai et al. 2013) enabling ultra-sensitive detection of bilirubin. Given the properties of UnaG, the bilirubin levels shown in the present study are likely to be strongly correlated with the total amount of bilirubin molecules that were present in the organelle during a certain period rather than with the bilirubin concentration at a specific time point.

Under dark conditions, we observed higher bilirubin accumulation in the stroma than under light conditions (**Fig. 1B**, **C**). This seems to contradict the fact that NADPH levels decrease during dark periods due to the arrest of photosynthesis (Lim et al. 2020). However, under light conditions, NADPH is consumed for CO_2_ assimilation, and the bilirubin produced is assumed to be degraded by the ROS generated by photosynthesis (Ishikawa et al. 2023). On the other hand, under dark conditions, NADPH is supplied by starch degradation and the oxidative pentose phosphorylation pathway (Backhausen et al. 1998). More bilirubin is produced in areas where starch is degraded, possibly leading to an uneven distribution of bilirubin within the stroma under dark conditions, as seen in **Fig. 1B**.

The data in **Fig. 2** show that bilirubin levels differed significantly between organelles, suggesting that bilirubin does not passively diffuse within cells. This is consistent with previous work showing that bilirubin is present at different concentrations in different organelles in animal cells (Park et al. 2016). On the other hand, a model was proposed in which bilirubin can pass through the membrane by a flip-flop mechanism (Zucker et al. 1999). In this model, bilirubin passes through the lipid bilayer membrane by flipping and then is carried by serum albumin to dissociate from the membrane. However, in the absence of serum albumin, the membrane permeability of bilirubin decreases significantly (Bulmer et al. 2008). Therefore, bilirubin does not appear to efficiently dissociate from the membrane and diffuse passively across membranes in cells containing little or no albumin. The chloroplast outer envelope surface might show a higher bilirubin level than the cytosol because bilirubin that is associated with the membrane was detected in addition to bilirubin in the cytosol (**Fig. 1B**, **C**).

Increased bilirubin synthesis within chloroplasts resulted in a decreased bilirubin level in peroxisomes (**Fig. 3**). We propose a model in which chloroplasts and peroxisomes actively exchange biliverdin and share the biliverdin pool. The ectopically expressed BVRA synthesizes bilirubin in chloroplasts from the biliverdin pool. Consequently, the amount of biliverdin converted to bilirubin in peroxisomes is expected to decrease. Peroxisomes are also rich in electron donors, including NADPH (Lim et al. 2020) and may produce bilirubin non-enzymatically. Another possibility is that heme is converted to biliverdin also in peroxisomes. The four *Arabidopsis* heme oxygenases have plastid transit peptides (Gisk et al. 2010), but if heme is catabolized to biliverdin by an unknown mechanism in peroxisomes, the ectopic expression of BVRA in plastids may reduce the heme supply to peroxisomes, resulting in reduced biliverdin and bilirubin production in peroxisomes. Although little is known about tetrapyrrole transport between organelles, this result implies the existence of an unknown tetrapyrrole transport pathway between plastids and peroxisomes.

Higher bilirubin level in root plastids relative to leaf chloroplasts (**Fig. 4**) likely represents a phenomenon similar to that seen in leaf chloroplasts under dark conditions. Root plastids do not generate large amounts of ROS from photosynthesis and they produce NADPH via the oxidative pentose phosphorylation pathway using starch as a carbon source. In fact, considerable NADPH and NADPH oxidase activity is detected in roots, as in leaves (Sgherri et al. 2002, Wang et al. 2018). Our findings imply that there is an intimate connection between bilirubin production and plastid functions other than photosynthesis.

A relatively large amount of bilirubin accumulated in the root plastids, but almost none accumulated in the amyloplasts in the root cap (**Fig. 4**). Similarly, in leaves, almost no bilirubin accumulated in the chloroplasts in guard cells. These observations suggest that differences in plastid functions lead to different levels of heme production and bilirubin accumulation. Future studies should be focused on the relationship between plastid functional differentiation and heme production and degradation.

## Materials and Methods

### Plant materials and growth conditions

We used *Arabidopsis* (*A. thaliana*) accession Columbia 0 (Col-0; CS60000) and *N. benthamiana* as the wild types. Seeds were surface-sterilized and sown on Murashige and Skoog (MS) medium containing 0.5% (w/v) gellan gum and 1% (w/v) sucrose. *Arabidopsis* seedlings were grown under continuous light (fluorescent tubes, 25 μmol m^−2^ s^−1^) at 22°C and were transferred to soil [potting mix (Nippi No.1 [Nihon Hiryo, Osaka, Japan]:vermiculite = 1:3] 11 d after sowing. *Nicotiana benthamiana* plants were grown under a 16 h-light/8 h-dark conditions at 25°C and were transferred to soil (potting mix:vermiculite = 1:3) 14 d after sowing.

### Plasmid construction and transformation

The *ptUnaG, TP-ratBVRA* and *TP-tagRFP* fragments were cloned into the pGWB602 (for *ptUnaG*) or pGWB511 (for *TP-ratBVRA* and *TP-tagRFP*) binary vector, which were designed to express the cloned genes under the control of the cauliflower mosaic virus (CaMV) *35S* promoter (Nakagawa et al. 2007) , as described previously (Ishikawa et al. 2023). All primers used for plasmid construction are listed in **Supplementary Table S1**. PCR-amplified DNA fragments and a synthetic DNA fragment were cloned into the pENTR1a vector (Thermo Fisher Scientific, Waltham, MA, USA.) at the SalI and EcoRV sites using the In-Fusion HD Cloning Kit (Clontech) according to the manufacturer’s instructions. The fragments cloned in pENTR1A were recombined into the pGWB602 binary vector using the Gateway LR reaction (Invitrogen). The DNA fragments encoding cytUnaG and poUnaG were amplified by PCR using pcDNA3–mCherry–UnaG as a template (Kumagai et al. 2013). The PCR fragments for poUnaG included a sequence encoding a peroxisome-targeting signal (SKL) at the C-terminus of mCherry–UnaG. For the cloning of *mtUnaG* and *oeUnaG*, DNA fragments that encode the N-terminal presequence [76 amino acid (aa) residues] of F1-ATPase γ-subunit (Lee et al. 2013) or the N-terminal sequence (50 aa residues) of *Arabidopsis* OUTER ENVELOPE PROTEIN7 (Oikawa et al. 2008) were amplified by PCR using Col-0 genomic DNA as a template. For the cloning of *tkUnaG*, the full-length coding sequence for *Arabidopsis BESTROPHIN-LIKE PROTEIN2* (Duan et al. 2016) was amplified by PCR using Col-0 genomic DNA as a template. Genomic DNA was extracted from Col-0 using the DNeasy Plant Mini Kit (Qiagen, Hilden, Germany) according to the manufacturer’s instructions. The two PCR products were joined by recombinant PCR with the fragment-encoding mCherry–UnaG, which was amplified from pcDNA3–mCherry–UnaG. For the cloning of *erUnaG*, a DNA fragment that encodes the N-terminal signal peptide (25 aa residues) of field pumpkin (*Cucurbita pepo*) 2S albumin was amplified from the genomic DNA of transgenic *Arabidopsis* plants expressing *SP-GFP-HDEL* (Matsushima et al. 2002). The fragments were joined with the *mCherry–UnaG* fragment by recombinant PCR with a primer containing sequences encoding the region encompassing the ER retention signal sequence histidine-aspartic acid-glutamic acid-leucine (HDEL). Transgenic *Arabidopsis* plants were generated using the floral dip method (Clough and Bent, 1998).

### Transient expression by agroinfiltration

Agrobacterium (*Rhizobium radiobacter*) strain GV2260 harboring pGWB602-cytUnaG, pGWB602-poUnaG, pGWB602-ptUnaG, pGWB602-mtUnaG, pGWB602-erUnaG, pGWB602-oeUnaG, pGWB602-tkUnaG, pGWB511-TP-ratBVRA or pGWB511-TP-tagRFP was cultured and resuspended in pure water to a final optical density of 1.0 at 600 nm. Single or mixed *Agrobacterium* cultures were syringe infiltrated into 4-week-old *N. benthamiana* leaves. The leaves were used for further experiments 2 d after infiltration.

### Confocal microscopy

Live-cell fluorescence imaging and measurement of bilirubin levels were performed primarily as described in our previous study (Ishikawa et al. 2023). In brief, images were captured with an SP8X confocal microscope system (Leica Microsystems, Wetzlar, Germany), equipped with Fluotar VISIR 25× and HC PL APO CS 63× water-immersion lenses. UnaG and mCherry were excited with 498- and 554-nm lasers from the white light laser source and detected at 510–545 and 565–636 by hybrid detectors, respectively. Chlorophyll autofluorescence was excited by a 554 nm laser and detected at 655–743 nm by a photomultiplier tube detector (Ishikawa et al. 2023). All images were taken in photon-counting mode using the time-gating method (Kodama, 2016). This method takes advantage of the short lifetime of chloroplast autofluorescence. By excluding the fluorescence emitted during 0.0–0.3 ns after excitation, chloroplast autofluorescence is suppressed below the detection limit and only the fluorescence of fluorescent proteins is specifically detected. Ratio metric images were output by dividing the fluorescence intensity of UnaG by the fluorescence intensity of mCherry at each pixel using Leica Application Suite X software. For the quantification of bilirubin levels, each circular ROI of a specific, defined size (plastid and cytosol, 5 × 5 pixels; the other organelles, 3 × 3 pixels) was randomly set on the target organelle in different cells, and fluorescence intensities of UnaG and/or mCherry were measured with Fiji software (Schindelin et al. 2012). Bilirubin levels upon TP-BVRA or TP-tagRFP coexpression (**Fig. 3**) were calculated as the mean value of UnaG fluorescence intensity. In the other experiments, bilirubin levels in tissues other than mesophyll cells or the root cortex of *Arabidopsis* were calculated using the following formula (Eq. 1):


(1) $${\mathrm{bilirubin\ level}}\, = \frac{1}{n}\mathop \sum \limits_{i = 1}^n \left( {{x_i}\frac{{\bar y}}{{{y_i}}}} \right),$$

where $x = \,$UnaG fluorescence intensity,$\,y = $ mCherry fluorescence intensity, $\bar y\, = \,$average mCherry fluorescence intensity and *n* = the number of ROI. For cotyledon mesophyll cells and the root cortex, we used the following formula (Eq. 2):


(2) $${\mathrm{bilirubin\ level}}\, = \frac{{{{\bar y}_{epi}}}}{{\bar y}}\left[ {\frac{1}{n}\mathop \sum \limits_{i = 1}^n \left( {{x_i}\frac{{\bar y}}{{{y_i}}}} \right)} \right]\; = \;\frac{1}{n}\mathop \sum \limits_{i = 1}^n \left( {{x_i}\frac{{{{\bar y}_{epi}}}}{{{y_i}}}} \right),$$

where ${\bar y_{epi}}\, = \,$average mCherry fluorescence intensity in the epidermal cells of cotyledons or roots (**Supplementary Figure S3E**).

## Supplementary Material

pcae017_Supp

## Data Availability

All data are available in the main text or the supplementary materials.
