# Supplementary material for: Bilirubin Distribution in Plants at the Subcellular and Tissue Levels
Source: Plant Cell Physiol. 2024 Feb 27;65(5):762–9. doi: 10.1093/pcp/pcae017 (PMC11138361; doi:10.1093/pcp/pcae017)
Supplement: pcae017_Supp [file pcae017_supp.zip › suppl_data/pcp-2024-e-00015-File008.pdf]

Figure S2, Ishikawa *et al.*

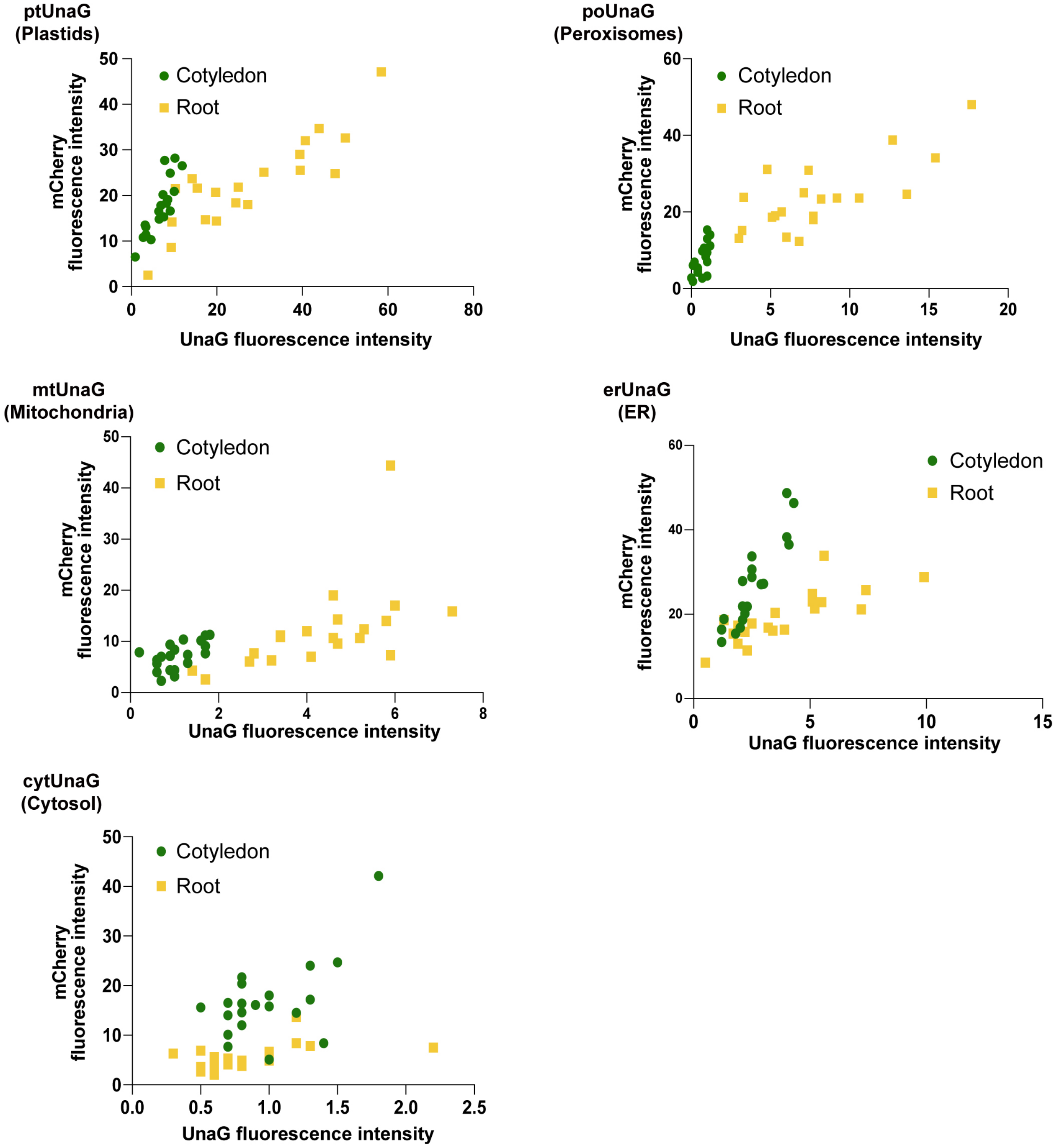

Figure S2. Scatterplots of UnaG and mCherry fluorescence intensity in each organelle.
