# Supplementary material for: Bilirubin Distribution in Plants at the Subcellular and Tissue Levels
Source: Plant Cell Physiol. 2024 Feb 27;65(5):762–9. doi: 10.1093/pcp/pcae017 (PMC11138361; doi:10.1093/pcp/pcae017)
Supplement: pcae017_Supp [file pcae017_supp.zip › suppl_data/pcp-2024-e-00015-File009.pdf]

Figure S3, Ishikawa *et al.*

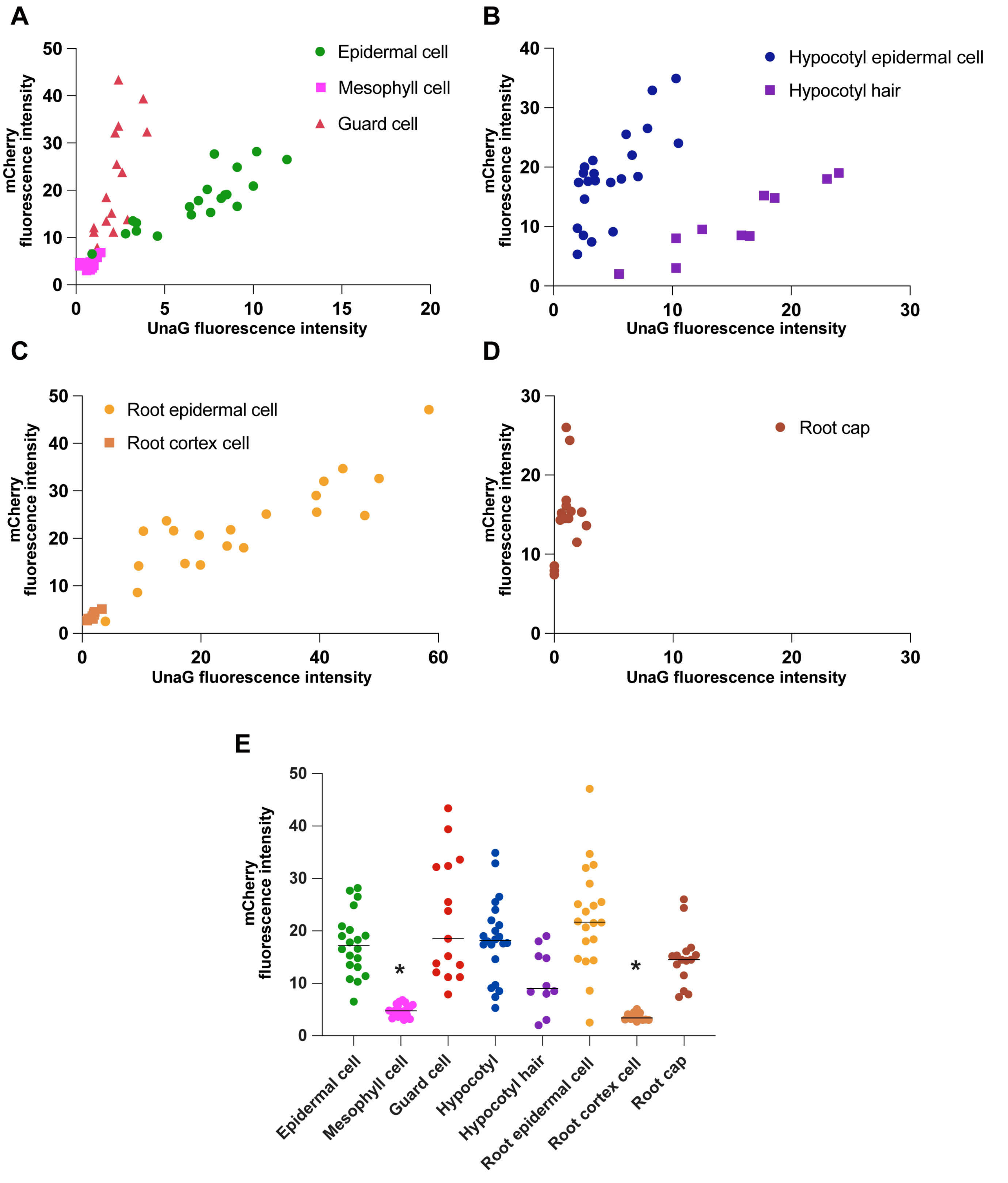

Figure S3. Analysis of UnaG and mCherry fluorescence intensity for the quantification of tissue bilirubin levels

(A–D) Scatterplots of UnaG and mCherry fluorescence intensity in plastids for each indicated tissue of 7-d-old Arabidopsis seedling expressing *ptUnaG*.

(E) Comparison of mCherry fluorescence intensity across tissues. Asterisks indicate significant differences relative to fluorescence from the cotyledon epidermis. $*P < 0.01$, using Dunnett's multiple comparisons test.
